# Biomechanical effects of orthodontic tooth movement on edentulous alveolar bone: a finite element analysis

**DOI:** 10.3389/fbioe.2025.1625027

**Published:** 2025-07-03

**Authors:** Xiaoxu Wang, Jiaojiao Xue, Xiaoting Wang, Mingyue Fan

**Affiliations:** Laboratory of Dental Biomaterials and Tissue Regeneration, Shanghai Xuhui District Stomatological Hospital, Shanghai, China

**Keywords:** maxillary central incisors, finite element analysis, orthodontic movement mechanics, alveolar bone remodeling, periodontal ligament stress

## Abstract

**Objective:**

This study investigates the biomechanical effects of different orthodontic movement patterns on the alveolar bone in the adjacent edentulous region through finite element analysis (FEA) of maxillary central incisor displacement mechanisms.

**Methods:**

A three-dimensional FEA model was constructed comprising the maxilla, upper dentition (with exclusion of the right maxillary central incisor), periodontal ligament (PDL), fixed orthodontic appliance bracket, and archwire system. The initial displacement characteristics and stress distribution patterns of the maxillary left central incisor (tooth 21) and the surrounding alveolar bone were quantitatively analyzed using ANSYS software.

**Results:**

Under an intrusion force approximately four times the lingual reactionary force, the maxillary central incisor underwent pure axial intrusion without labial or lingual tipping. Adjacent tooth movement had minimal mechanical impact on the edentulous alveolar bone (<0.5% strain). The resistance center of the incisor was identified 0.43 times the root length apical to the alveolar ridge crest, necessitating precise force vector alignment to achieve bodily movement without rotational displacement or alveolar bone remodeling. For incisal tipping correction, a horizontal tensile force (F) combined with an archwire-bracket-induced moment (M) allows controlled tipping via modulation of the M/F ratio. However, excessive force risks pathological root resorption and alveolar bone atrophy in edentulous regions.

**Conclusion:**

Bodily movement of the central incisor, when guided through the resistance center, does not significantly remodel the edentulous alveolar bone. Moreover, effective tipping correction requires precise M/F ratio control to optimize movement efficiency while minimizing iatrogenic risks. Deviations from optimal force parameters substantially increase the likelihood of alveolar bone atrophy.

## 1 Introduction

Prolonged edentulism in the anterior maxilla poses significant restorative challenges, often leading to mesial drift of adjacent teeth, loss of prosthetic space, alveolar ridge defects, and progressive soft tissue atrophy (Wright et al., 2016). These structural changes not only compromise implant placement but also create functional and aesthetic limitations, making rehabilitation increasingly complex over time. Multidisciplinary preprosthetic orthodontic intervention has become as a standardized protocol to mitigating these effects, addressing three fundamental objectives: (1) optimizing dental arch space to ensure proper prosthetic integration, (2) stimulating periodontal ligament (PDL) adaptation to enhance alveolar bone volume and density, and (3) remodeling compromised hard and soft tissues to establish a more favorable foundation for aesthetic reconstruction (Isola et al., 2016a). However, despite advancements in orthodontic techniques, predicting the precise biomechanical impact of tooth movement on alveolar bone remains a challenge. The interplay between force magnitude, direction, and duration in influencing bone remodeling patterns has yet to be fully elucidated, particularly in edentulous regions where physiological responses may differ from those of dentate sites (Ammoury MJ. et al., 2019).

Traditional clinical and radiographic assessments provide valuable insights into orthodontic treatment outcomes but are inherently limited in their ability to capture the complex, dynamic interactions between mechanical forces and biological tissue responses (Ozdemir et al., 2013). *In vivo* studies are constrained by ethical considerations, patient variability, and the difficulty of measuring localized stress distributions within bone structures. Finite Element Analysis (FEA) offers a powerful alternative by enabling a controlled, non-invasive simulation of orthodontic biomechanics (Ammoury MJ. et al., 2019; Elshazly et al., 2023). This computational technique models stress-strain distributions within three-dimensional (3D) structures, allowing for precise evaluation of tissue-level responses to applied forces (Ghafari and Ammoury, 2020). By simulating various loading conditions, FEA provides a predictive framework for understanding how different tooth movement modalities influence alveolar bone remodeling, helping to refine treatment protocols for improved clinical outcomes (Wegst and Ashby, 2004).

Recent advances in FEA modeling have significantly enhanced its clinical applicability (de Brito et al., 2019). The integration of high-resolution imaging, patient-specific anatomical reconstructions, and refined material property datasets has improved the accuracy of stress distribution predictions within bone and PDL structures (Mattu et al., 2021). Building upon this foundation, the present study develops a comprehensive 3D dentition-maxilla-orthodontic appliance-archwire system model to systematically investigate the biomechanical effects of anterior tooth movement on edentulous ridge bone volume. This approach aims to bridge the gap between theoretical predictions and clinical outcomes, providing a deeper understanding of the underlying mechanical principles governing orthodontic treatment in compromised alveolar environments (Yang et al., 2013).

To build upon this foundation, the present study systematically evaluates the biomechanical effects of different orthodontic movement modes on the alveolar bone within an adjacent edentulous region (Hahn W. et al., 2010). By employing a refined 3D finite element model, we aim to quantify stress distribution and displacement characteristics under controlled orthodontic force applications. This analysis will provide critical insights into the mechanical responses of alveolar bone and periodontal structures, allowing for a deeper understanding of force optimization strategies (Hahn et al., 2009). The following sections detail the methodology used to construct and validate the finite element model, the experimental configurations for different orthodontic movement patterns, and the resulting biomechanical implications for clinical orthodontic treatment planning.

## 2 Materials and methods

### 2.1 Instruments and equipment

The finite element analysis (FEA) in this study utilized Mimics 21.0 for 3D anatomical reconstruction, Geomagic 2021 for surface refinement, SolidWorks 2017 for CAD-based modeling of orthodontic appliances, and Ansys Workbench 2022 for mesh generation and biomechanical simulations. This integrated workflow ensured high-precision modeling, allowing for an accurate assessment of stress distribution and displacement within the alveolar bone and periodontal structures.

### 2.2 Establishment of a coordinate system

For this finite element analysis, a 30-year-old male volunteer with a fully developed permanent dentition and a normal, well-aligned maxillary and mandibular arch was selected as the reference subject. The individual exhibited no signs of caries, root inflammation, or occlusal abnormalities, ensuring a representative and clinically relevant model. To create the 3D model, the DICOM file images were imported and segmented with the use of Mimics 21.0, where segmentation was performed to isolate the teeth, cortical and cancellous bone, and maxillary structures. Then, the model was modified with Geomagic 2021 for surface smoothing and denoising followed by SolidWorks 2017, where orthodontic components, including the archwire and brackets, were precisely designed. The bracket groove, measuring 0.022 × 0.028 inches, was positioned at the buccal center of the clinical crown, parallel to the occlusal plane (Ammoury MJ. et al., 2019). To accurately represent periodontal dynamics, a periodontal ligament (PDL) shell was generated by expanding the root boundary outward by 0.25 mm, ensuring a realistic simulation of biomechanical interactions.

The finalized model was imported into Ansys Workbench 2022, where mesh generation and constraint application were performed. A ten-node tetrahedral mesh was generated, consisting of 518,690 nodes and 295,247 elements, ensuring a balance between computational efficiency and anatomical accuracy. Material properties, including elastic modulus and Poisson’s ratio, were assigned based on established literature values. All biomaterials were modeled as continuous, homogeneous, isotropic linear elastomers, with the alveolar bone base serving as a fixed boundary condition to replicate physiological constraints (Ghafari and Ammoury, 2020). The elasticity modulus of the different constituent units of the finite element model is shown in Table 1 (Wegst and Ashby, 2004; de Brito et al., 2019).

**TABLE 1:** Composition of finite element model and set values of its elasticity modulus.

Material	Elasticity modulus (MPa)	Poisson’s ratio
Cortical bone	13,700	0.30
Cancellous bone	1,370	0.30
Teeth	20,700	0.30
Periodontal ligament	68.9	0.45
Bracket	206,000	0.30
Stainless steel archwire	176,000	0.30
NiTi archwire	60,000	0.30
Australian archwire	173,000	0.30

### 2.3 Confriguration setting

**FIGURE 1:**
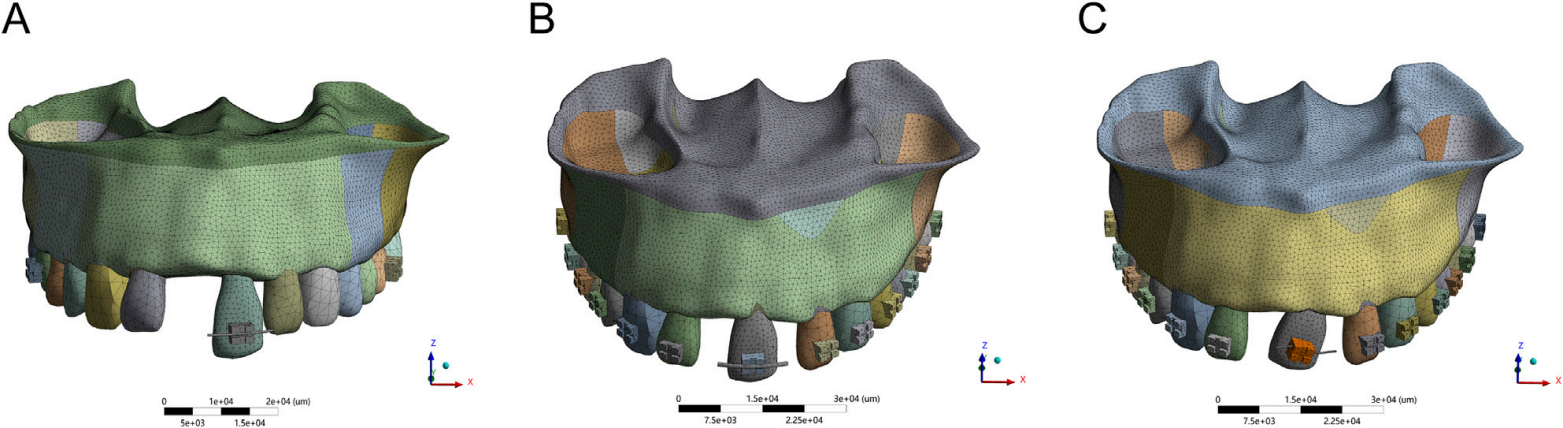
The axial extrusion **(A)**, mesial translation **(B)** and mesial tipping of 3D finite analysis **(C)**.

To investigate the biomechanical effects of orthodontic tooth movement on the adjacent atrophic alveolar ridge, three standardized displacement configurations were designed: axial intrusion, mesial translation, and mesial tipping. These movement types are visually presented in Figure 1, which illustrates the corresponding vectors applied to Tooth 21 in a simulated 3D finite element model.

Axial intrusion was used to simulate pure vertical displacement of the maxillary left central incisor (Tooth 21) along its longitudinal axis. A controlled 3 mm displacement was applied while maintaining crown-root angulation to ensure uniform force distribution. The effect of this movement on alveolar bone height was measured from the cementoenamel junction (CEJ), providing insights into potential ridge morphology changes.

Mesial translation replicated bodily movement of Tooth 21 in the mesial direction over a 4 mm displacement. To maintain periodontal ligament (PDL) integrity and prevent excessive stress concentration, PDL width was preserved above 1.5 mm throughout the movement. A right-handed coordinate system was established, with the X-axis representing mesial-distal movement, the Y-axis indicating sagittal displacement, and the Z-axis corresponding to vertical displacement.

Mesial tipping was applied to evaluate rotational displacement, with a 20° rotation around the apical third of the root to replicate clinical tipping mechanics. The moment arm was set to maintain a 1:1 crown-root ratio, ensuring controlled movement while preventing excessive stress on the alveolar ridge. Additionally, a 0.5 mm gingival displacement threshold was introduced to simulate the potential impact on soft tissue structures. The axial intrusion, mesial translation and mesial tipping are displaced respectively in Figure 1.

### 2.4 Loading method

The main archwire was positioned within the bracket groove, with a nonlinear spring element connecting the bracket and archwire to replicate passive ligation in clinical practice. This setup fully constrained separation between the archwire and bracket while allowing unrestricted approximation. During calculations, the straight-wire brackets maintained direct contact with the tooth surface to ensure accurate force transmission. Orthodontic forces were applied according to the three displacement configurations, as detailed in Table 2. Tooth movement and alveolar bone displacement were analyzed using displacement moiré patterns, where color bars consistently represented displacement magnitudes. Stress concentration areas were evaluated through periodontal ligament and alveolar bone stress distribution diagrams, providing insights into biomechanical responses under each loading condition.

**TABLE 2:** Constraints and application of orthodontic force.

Configuration	Constraints	Application position	Loading protocols
Axial Intrusion	Axial fixation constraints​ were imposed at both ends of the archwire	Center point of the bracket groove (gingival side)	① Pure axial load: 15 g (0.147 N) along the tooth’s long axis ② Controlled tipping resistance: 15 g (0.147 N) + 0.5 N mm anti-labial moment (Mp) to maintain crown-root alignment
Mesial Translation	• Axial fixation at both ends of the archwire • Mesial wing movement restricted in Y-direction	• Center point of the distal wing of the bracket • Distal wing point D of the long draw hook	① Bodily translation: 50 g (0.49 N) tensile force applied distally through the bracket center ② Enhanced control: 50 g (0.49 N) + optimal lever arm (Db = 15 mm) through point D to minimize rotational displacement
Mesial Tipping	• Axial fixation at both ends of the archwire • Free movement allowed for Tooth 21 only	① A counterclockwise couple applied at the bracket center ② ​A counterclockwise couple applied at the bracket center + tensile force F at the distal wing of the bracket	① Initial tipping: 1 N mm counterclockwise couple applied at the bracket center ② Dynamic uprighting: 1 N mm counterclockwise couple + optimal tensile force Ft distally through the bracket to achieve controlled root apex rotation and alignment

### 2.5 Alveolar bone displacement measurement protocol

Four key observation points were selected to assess alveolar bone displacement: (a) the center of the alveolar ridge apex on the labial side of Tooth 21, (b) the point of maximum stress near the root on the labial side of the edentulous region, (c) the center of the alveolar ridge apex in the edentulous region, and (d) the center of the alveolar ridge apex on the mesial surface of Tooth 21. Displacement in three dimensions was quantified for each point using Ansys Workbench 2022 under all specified configurations.

## 3 Results

### 3.1 Axial intrusion produces controlled vertical displacement and stress redistribution

**FIGURE 2:**
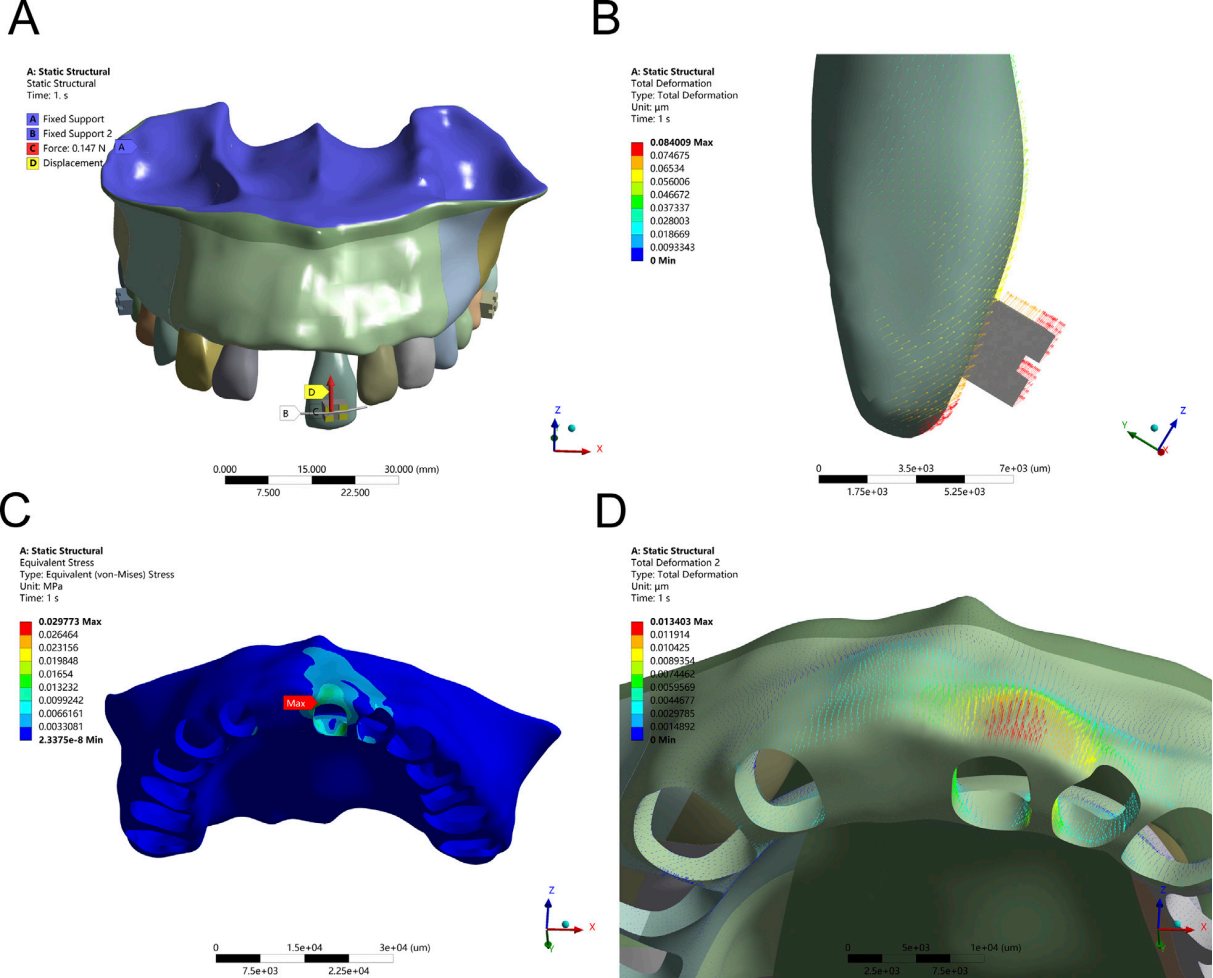
Intrusion Configuration. **(A)** Application of a 15 g intrusion force along the long axis of the tooth. **(B)** Displacement pattern of Tooth 21 under applied force. **(C)** Maximum alveolar bone stress (0.0298 MPa) concentrated at the labial ridge crest. **(D)** Greatest alveolar bone displacement observed on the labial side of Tooth 21.

**FIGURE 3:**
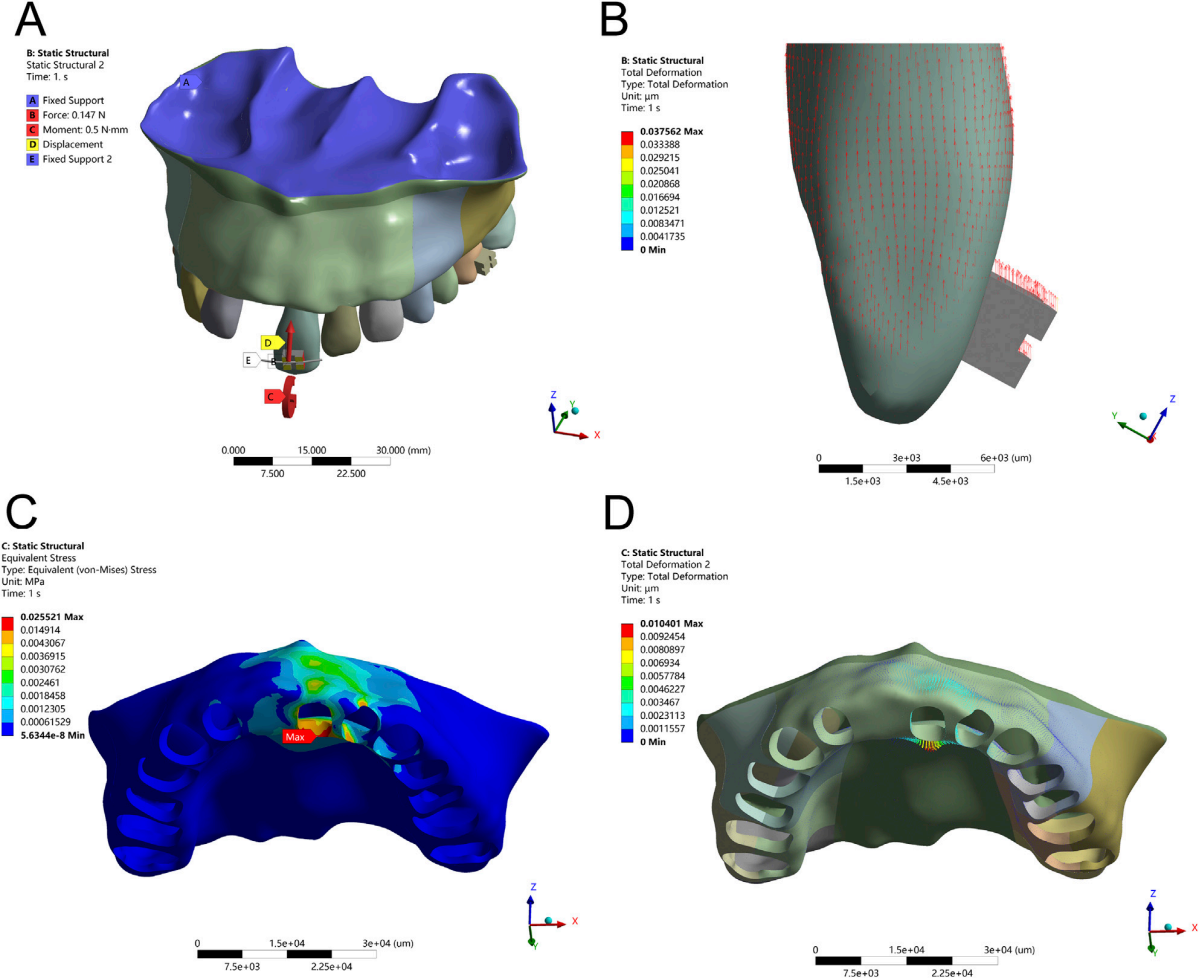
Axial Intrusion Configuration – **(A)** 15 g intrusion force applied along the tooth’s long axis with an anti-labial tipping moment (M) for controlled movement. **(B)** Displacement pattern of Tooth 21 following force application. **(C)** Peak alveolar bone stress (0.0255 MPa) localized at the labial ridge crest. **(D)** Greatest displacement observed on the lingual side of the alveolar bone.

Finite element analysis of axial intrusion revealed that applying a 15 g intrusive force along the long axis of Tooth 21 produced controlled vertical displacement, with maximum PDL stress localized at the root apex (9.12 kPa) and alveolar bone stress peaking at the labial ridge crest (0.0298 MPa). However, despite this controlled vector, a slight forward tipping of the crown was observed due to unopposed force imbalance. The stress distribution and displacement patterns under simple axial intrusion and optimized force application are shown in Figures 2, 3 and Table 3, respectively.

**TABLE 3:** Comparison of displacements (μm) at each observation point under intrusion load (E^-X^ for E^-X^).

Observation point	15 g intrusion load along the long axis of the tooth				15 g intrusion load along the long axis of the tooth + optimal anti-labial tipping moment Mp			
	Total displacement	X-axis	Y-axis	Z-axis	Total displacement	X-axis	Y-axis	Z-axis
a	1.340E-2	3.359E-3	−8.438E-3	9.856E-3	1.040E-2	8.555E-5	9.426E-3	−4.935E-3
b	2.953E-3	−3.394E-4	−7.972E-4	2.823E-3	6.133E-4	−5.164E-5	1.655E-4	5.883E-4
c	5.143E-3	1.75E-3	−2.699E-3	4.013E-3	1.559E-3	8.051E-4	7.093E-4	1.132E-3
d	7.210E-3	1.809E-3	−3.309E-3	6.146E-3	2.630E-3	6.535E-4	1.260E-3	2.215E-3

To counteract this labial inclination, a compensatory moment of 0.5 Nmm was introduced. This adjustment refined the movement vector, promoting pure axial displacement. Following this intervention, stress within the PDL became more uniformly distributed, and peak bone stress at the labial ridge crest decreased to 0.0255 MPa. These changes confirm that fine-tuning the force-to-moment ratio is crucial for eliminating rotational components and enhancing the predictability of vertical tooth movement without jeopardizing periodontal stability.

### 3.2 Mesial translation influences bodily movement and alveolar bone stress

**FIGURE 4:**
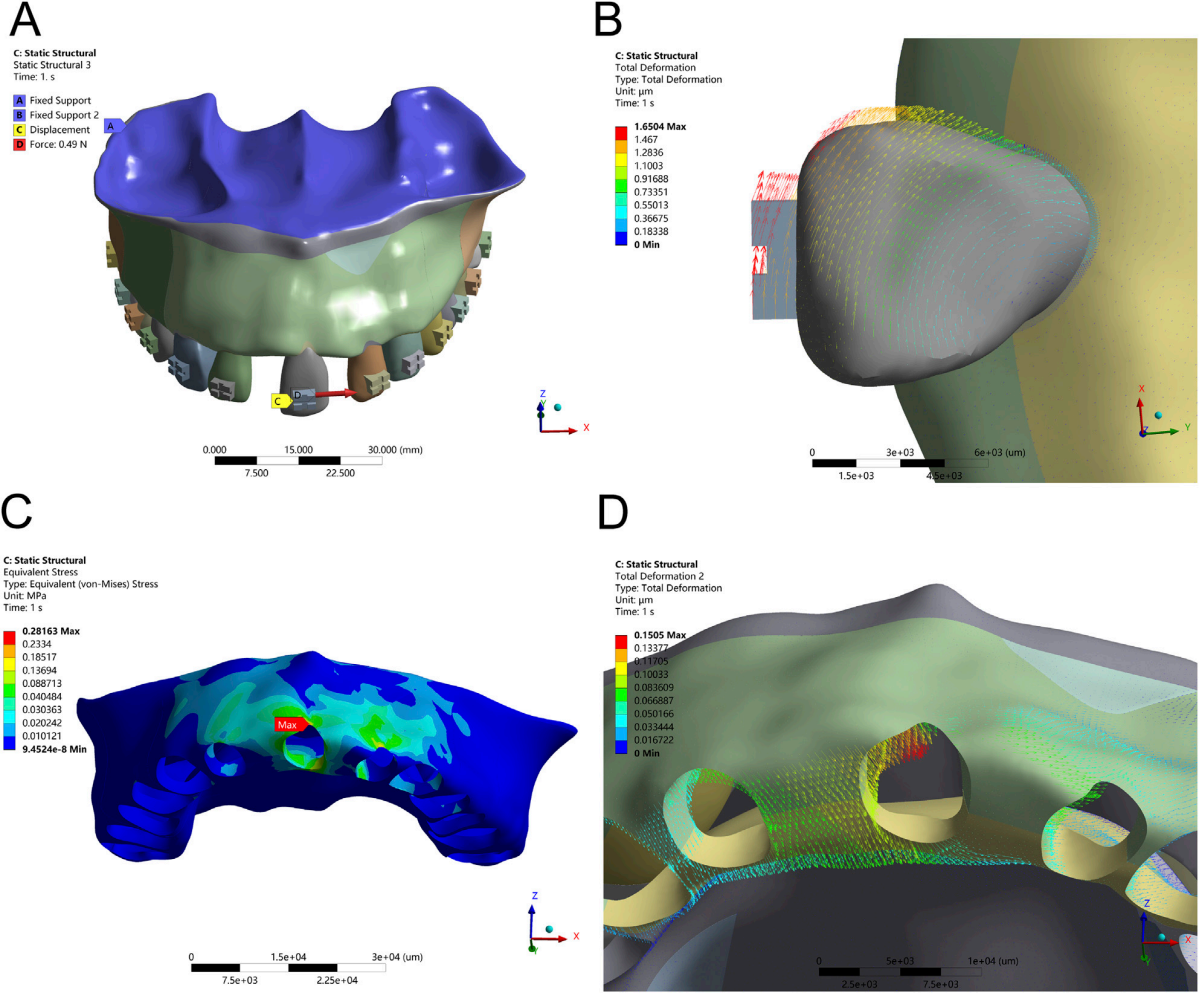
Translation Configuration – **(A)** Application of a 50 g tensile force from the bracket in the distal direction. **(B)** Displacement pattern of Tooth 21 under mesial translation. **(C)** Peak alveolar bone stress (0.2816 MPa) concentrated at the labial ridge. **(D)** Greatest alveolar bone displacement observed on the mesial labial side of Tooth 21.

**FIGURE 5:**
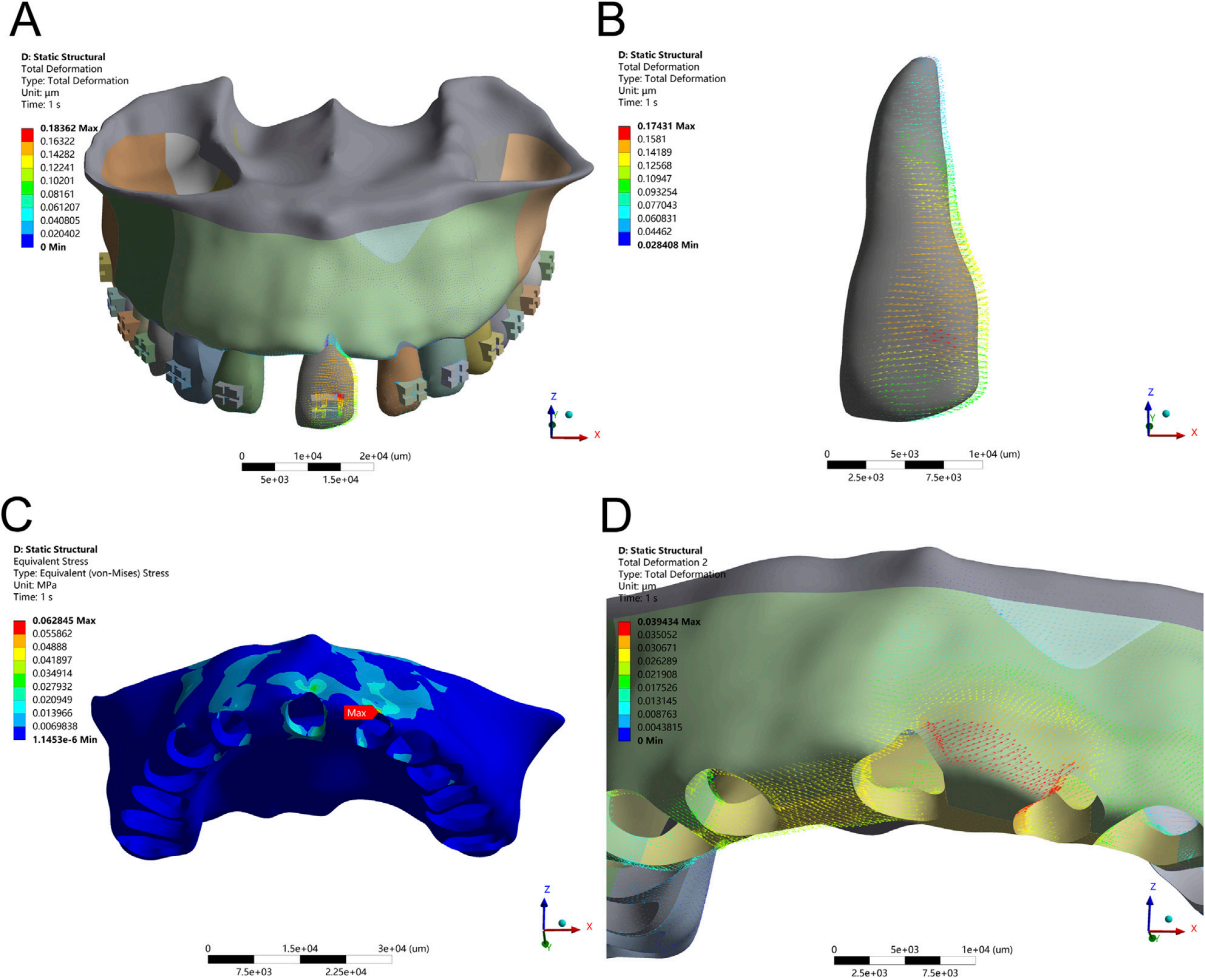
Translation Configuration – **(A)** Application of a 50 g tensile force from point D of the long draw hook in the distal direction, using an optimized force arm length (Db). **(B)** Displacement pattern of Tooth 21 under controlled translation. **(C)** Peak alveolar bone stress (0.0628 MPa) distributed along the ridge. **(D)** Maximum displacement observed on the distal side of Tooth 21.

In the mesial translation simulation, a 50 g tensile force applied to the distal bracket wing caused disproportionate movement of the crown compared to the root apex. The crown exhibited a displacement of 0.32 mm, while the root apex moved only 0.08 mm, resulting in uncontrolled tipping rather than the intended bodily translation. This asymmetrical movement led to high PDL stress at the root apex (40 kPa) and pronounced alveolar bone stress at the labial crest (0.2816 MPa), posing potential risks for localized bone remodeling or damage. The biomechanical effects of mesial translation, both with direct distal force and with an optimized force arm, are depicted in Figures 4, 5 and Table 4.

**TABLE 4:** Comparison of Displacements (μm) at each Observation Point under Translation force (E-X for E-X).

Observation point	50 g tensile force from the bracket to the distal direction				50 g tensile force from point D of the long draw hook to the distal direction, with the optimal force arm length of Db			
	Total displacement	X-axis	Y-axis	Z-axis	Total displacement	X-axis	Y-axis	Z-axis
a	1.505E-1	6.505E-2	1.135E-1	−7.438E-2	3.943E-2	3.492E-2	−1.305E-2	1.285E-2
b	5.964E-2	3.191E-2	2.156E-2	−4.554E-2	2.135E-2	1.845E-2	4.482E-3	9.762E-3
c	8.285E-2	4.177E-2	4.750E-2	−5.351E-2	2.746E-2	2.577E-2	3.521E-3	8.793E-3
d	9.112E-2	4.442E-2	5.570E-2	−5.681E-2	2.718E-3	2.618E-2	3.777E-3	6.224E−3

To achieve true bodily movement, an optimized long-draw hook with a force arm length of 3.19 mm was integrated. This configuration significantly improved movement alignment between the crown and root, reflected by a reduction in alveolar bone stress to 0.0628 MPa and a more evenly distributed PDL stress pattern (peak 6.4 kPa). These results emphasize the biomechanical advantage of incorporating auxiliary mechanics to regulate the M/F ratio and avoid unwanted rotational tendencies during translational tooth movement.

### 3.3 Mesial tipping alters root apex rotation and ridge crest stress

**FIGURE 6:**
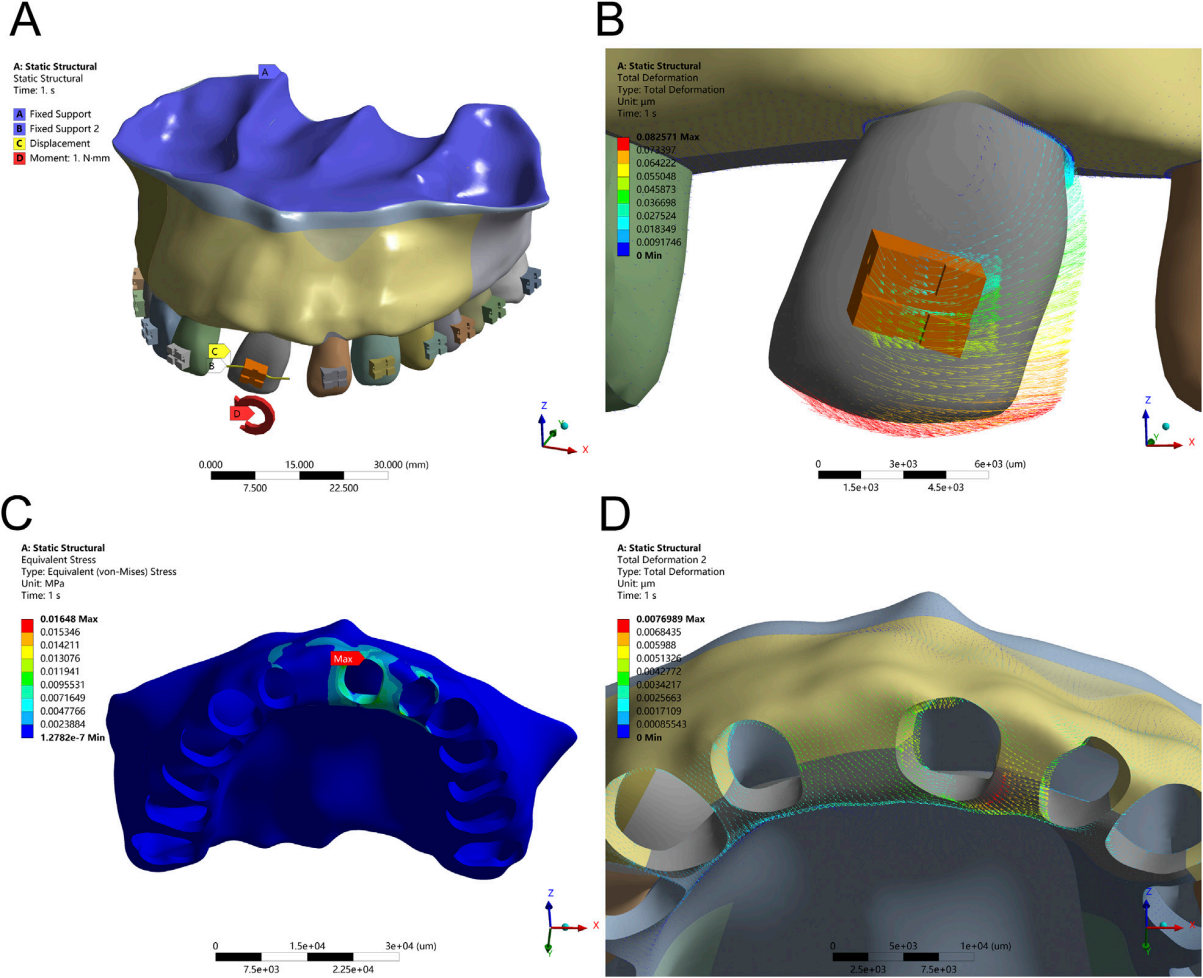
Tipping Configuration – **(A)** Application of a 1 Nmm counterclockwise couple moment at the bracket center. **(B)** Displacement pattern of Tooth 21 under tipping mechanics. **(C)** Peak alveolar bone stress (0.0165 MPa) concentrated along the ridge. **(D)** Maximum displacement of Tooth 21 at the distal alveolar bone.

**FIGURE 7:**
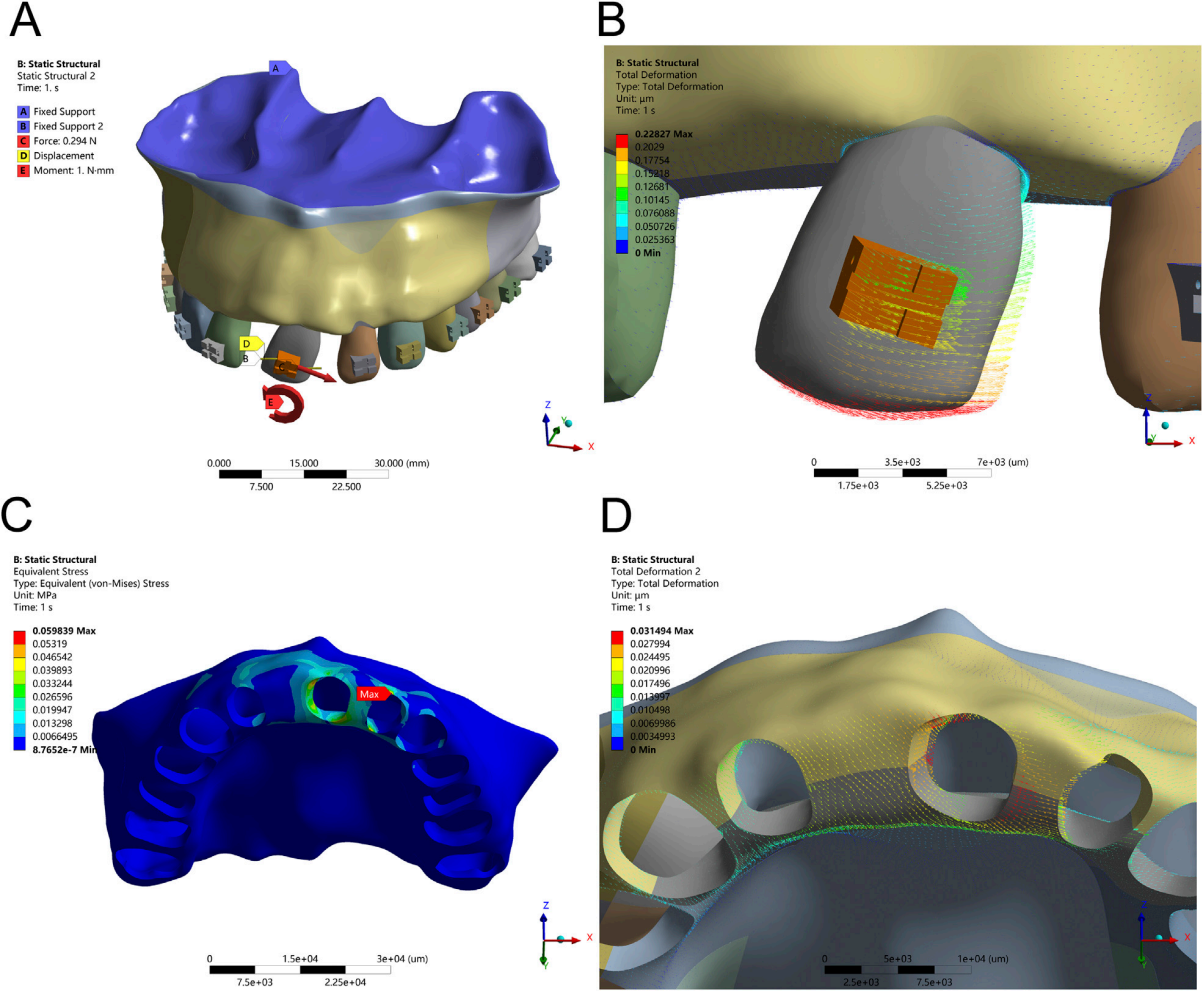
Tipping Configuration – **(A)** Application of a 1 Nmm counterclockwise couple moment at the bracket center, combined with an optimal tensile force (Ft) from the distal bracket wing in the distal direction. **(B)** Displacement pattern of Tooth 21 under optimized tipping mechanics. **(C)** Peak alveolar bone stress (0.0598 MPa) concentrated along the ridge. **(D)** Maximum displacement observed at the mesial and distal alveolar ridge crests.

The application of a counterclockwise couple (1 Nmm) at the bracket center simulated clinical mesial tipping. Initial results indicated a torsional rotation centered around the apical third of the root, with crown displacement measuring 0.35 mm and root apex displacement limited to 0.08 mm. PDL stress remained low (0.0165 MPa), and alveolar bone stress was localized at the mesial and distal crests, suggesting effective but limited control over the rotational center. Mesial tipping mechanics, with and without auxiliary tensile force, are illustrated in Figures 6, 7 and Table 5, respectively.

**TABLE 5:** Comparison of Displacements (μm) at each Observation Point under a Couple of Moments (E-X for E-X).

Observation point	Counterclockwise couple moments at the center point of the bracket, M = 1 N·mm				Counterclockwise couple moment of 1 N·mm at the center point of the bracket + optimal tensile force Ft from the distal wing of the bracket to the distal direction			
	Total displacement	X-axis	Y-axis	Z-axis	Total displacement	X-axis	Y-axis	Z-axis
a	7.699E-3	5.913E-3	2.390E-3	−4.313E-3	3.149E-2	1.725E-2	1.923E-2	−1.801E-2
b	2.084E-3	4.657E-4	1.381E-4	−2.026E-3	1.601E-2	7.780E-3	2.586E-3	−1.314E-2
c	3.537E-3	2.498E-3	1.022E-3	−2.287E-3	2.191E-2	1.504E-2	7.841E-3	−1.386E-2
d	4.246E-3	3.145E-3	5.672E-4	−2.795E-3	2.546E-2	1.744E-2	7.231E-3	−1.709E-2

To enhance root apex guidance and improve the crown-to-root displacement balance, an additional 30 g distal tensile force was applied. This adjustment increased the crown-to-root movement ratio to 2.1:1 and slightly elevated PDL stress to 0.0341 MPa—still within safe biomechanical thresholds. Alveolar bone stress remained controlled (peak 0.0598 MPa), indicating that combined couple and tensile loading provides enhanced control over rotational displacement, minimizing undesired stress concentrations in adjacent edentulous areas.

## 4 Discussion

This study used finite element analysis to investigate the biomechanical effects of different orthodontic movement modalities on adjacent edentulous alveolar bone. The simulations revealed that subtle variations in force application and moment control significantly influence stress distribution within both the periodontal ligament (PDL) and alveolar bone, particularly in compromised anterior maxillary regions.

Axial intrusion, although typically stable, produced labial tipping when the force vector did not pass through the tooth’s resistance center. Introducing a compensatory moment eliminated this tendency, restoring pure vertical displacement and balancing stress across the PDL. These findings align with prior experimental studies emphasizing the critical role of moment-to-force (M/F) ratio optimization in achieving bodily movement without overloading apical tissues (Kusy and Tulloch, 1986; Liang et al., 2008). In clinical settings, failure to control such vectors may exacerbate bone remodeling in atrophic ridges and compromise implant site stability (Zhao et al., 2007; Machado et al., 2010).

During mesial translation, unadjusted tensile force resulted in rotational displacement and excessive stress at the labial alveolar crest. The incorporation of a 3.19 mm long-draw hook created a more favorable M/F ratio, aligning crown and root movement and significantly reducing stress concentrations. Although this parameter yielded favorable results in our model, further research is needed to evaluate alternative force arm lengths across varying anatomical configurations. Recent modeling studies similarly highlight the mechanical advantage of extended force arms or auxiliary devices in achieving true bodily tooth movement (Ammoury L. A. et al., 2019; Gomez et al., 2015). These findings align with previous reports on the importance of force arm length in minimizing stress and improving tooth movement precision (Kusy and Whitley, 1991; Orth et al., 2007).

In mesial tipping, the application of a couple moment alone resulted in limited root control. When combined with a 30 g tensile force at the distal bracket wing, the rotation center shifted apically, producing a more balanced crown-to-root displacement and maintaining PDL stress within physiological limits. This reflects a broader trend in aligner and bracket-based mechanics where the controlled application of couples and traction improves predictability in tipping scenarios (Hahn W. M. et al., 2010; Li et al., 2013). However, defining a universally “optimal” tensile force requires further empirical validation (Zeng et al., 2017).

Interestingly, across all movement modes, even indirect mechanical loading appeared to influence the edentulous ridge, albeit at low strain levels (Isola et al., 2016b). While unlikely to cause remodeling in isolation, such secondary strain propagation supports recent mechanobiological findings that bone adaptation is influenced not just by peak stresses but also by strain frequency and distribution over time (Ma et al., 2019; Becker et al., 2006). This may hold implications for patients undergoing prolonged orthodontic preparation prior to prosthetic restoration.

Overall, the study confirms the importance of biomechanical precision in orthodontic treatment planning, particularly when working near structurally vulnerable areas. Proper M/F control, force vector alignment, and auxiliary mechanics are critical for minimizing unintended stress accumulation and enhancing movement efficiency. While the current model used idealized material properties and a single anatomy, future work should incorporate patient-specific geometries and nonlinear tissue behavior to refine clinical recommendations. Finite element modeling remains a valuable predictive tool for assessing complex movement scenarios, offering insights that can improve safety and outcomes in interdisciplinary orthodontic care (Misch, 2015).

## 5 Conclusion

This study demonstrates that controlled axial intrusion minimizes unwanted tipping, mesial translation requires force arm optimization for bodily movement, and mesial tipping benefits from precise moment-to-force (M/F) ratio adjustments to stabilize root apex displacement. Effective force vector control and strategic force modulation enhance movement efficiency while reducing alveolar bone stress and periodontal risk. Incorporating patient-specific anatomical variations and long-term clinical validation will further refine these biomechanical principles for improved orthodontic treatment planning.

## Data Availability

The original contributions presented in the study are included in the article/supplementary material, further inquiries can be directed to the corresponding author.
